# Supplementary material for: Meta-Analysis and Gene Set Enrichment Relative to ER Status Reveal Elevated Activity of MYC and E2F in the “Basal” Breast Cancer Subgroup
Source: PLoS One. 2009 Mar 9;4(3):e4710. doi: 10.1371/journal.pone.0004710 (PMC2650420; doi:10.1371/journal.pone.0004710)
Supplement: Dataset S1 — GSEA results from first screen. The file contains a zipped archive of the HTML results of the second GSEA screen. Reports for enriched or depleted datasets may be accessed from the “index.html” link within the my_analysis.GseaPreranked.1219975950625.rpt.zip ZIP archive. Enrichment plots are available for each of the 50 most enriched and depleted gene sets. (13.32 MB ZIP) [file pone.0004710.s001.zip › my_analysis.GseaPreranked.1219975950625.rpt.zip!//BRCA_BRCA1_POS.html]

Details for gene set BRCA\_BRCA1\_POS[GSEA]

|  || Dataset | zSco.ERneg.to.ERpos\_collapsed |
| Phenotype | NoPhenotypeAvailable |
| Upregulated in class | na\_pos |
| GeneSet | BRCA\_BRCA1\_POS |
| Enrichment Score (ES) | 0.66533077 |
| Normalized Enrichment Score (NES) | 2.689827 |
| Nominal p-value | 0.0 |
| FDR q-value | 0.0 |
| FWER p-Value | 0.0 |
Table: GSEA Results Summary

  

Fig 1: Enrichment plot: BRCA\_BRCA1\_POS      
 Profile of the Running ES Score & Positions of GeneSet Members on the Rank Ordered List

  

| PROBE | GENE SYMBOL | GENE\_TITLE | RANK IN GENE LIST | RANK METRIC SCORE | RUNNING ES | CORE ENRICHMENT || 1 | SLC43A3 |  |  | 10 | 7.126 | 0.0256 | Yes |
| 2 | VGLL1 |  |  | 18 | 6.865 | 0.0505 | Yes |
| 3 | LMO4 |  |  | 23 | 6.717 | 0.0751 | Yes |
| 4 | KCNN4 |  |  | 54 | 6.289 | 0.0961 | Yes |
| 5 | NMB |  |  | 71 | 6.063 | 0.1173 | Yes |
| 6 | SOX10 |  |  | 107 | 5.831 | 0.1363 | Yes |
| 7 | YBX1 |  |  | 157 | 5.523 | 0.1530 | Yes |
| 8 | ZNF259 |  |  | 167 | 5.482 | 0.1726 | Yes |
| 9 | PSME4 |  |  | 175 | 5.460 | 0.1923 | Yes |
| 10 | MCM4 |  |  | 180 | 5.436 | 0.2121 | Yes |
| 11 | D21S2056E |  |  | 195 | 5.326 | 0.2308 | Yes |
| 12 | REXO2 |  |  | 234 | 5.170 | 0.2471 | Yes |
| 13 | LRRC8D |  |  | 237 | 5.158 | 0.2660 | Yes |
| 14 | LAMP3 |  |  | 247 | 5.108 | 0.2842 | Yes |
| 15 | ZRF1 |  |  | 432 | 4.585 | 0.2873 | Yes |
| 16 | MPZL1 |  |  | 449 | 4.518 | 0.3028 | Yes |
| 17 | ART3 |  |  | 450 | 4.517 | 0.3195 | Yes |
| 18 | KIAA0020 |  |  | 467 | 4.469 | 0.3349 | Yes |
| 19 | SLC5A6 |  |  | 474 | 4.453 | 0.3509 | Yes |
| 20 | PPP1CB |  |  | 533 | 4.274 | 0.3623 | Yes |
| 21 | SFT2D2 |  |  | 544 | 4.253 | 0.3773 | Yes |
| 22 | WDR43 |  |  | 578 | 4.189 | 0.3903 | Yes |
| 23 | DDX21 |  |  | 653 | 4.024 | 0.3996 | Yes |
| 24 | UCHL3 |  |  | 667 | 4.000 | 0.4135 | Yes |
| 25 | GEMIN4 |  |  | 672 | 3.988 | 0.4279 | Yes |
| 26 | GLMN |  |  | 716 | 3.904 | 0.4391 | Yes |
| 27 | CALB2 |  |  | 736 | 3.867 | 0.4520 | Yes |
| 28 | CEP57 |  |  | 739 | 3.862 | 0.4662 | Yes |
| 29 | SERBP1 |  |  | 742 | 3.856 | 0.4803 | Yes |
| 30 | UCHL5 |  |  | 765 | 3.814 | 0.4927 | Yes |
| 31 | C1ORF38 |  |  | 850 | 3.705 | 0.5001 | Yes |
| 32 | KIAA0179 |  |  | 885 | 3.648 | 0.5110 | Yes |
| 33 | ATF4 |  |  | 959 | 3.540 | 0.5186 | Yes |
| 34 | PDXK |  |  | 961 | 3.540 | 0.5317 | Yes |
| 35 | MTMR2 |  |  | 965 | 3.531 | 0.5445 | Yes |
| 36 | DDX39 |  |  | 989 | 3.498 | 0.5557 | Yes |
| 37 | INDO |  |  | 995 | 3.489 | 0.5682 | Yes |
| 38 | MYC |  |  | 1025 | 3.447 | 0.5788 | Yes |
| 39 | RPL5 |  |  | 1050 | 3.410 | 0.5896 | Yes |
| 40 | SOD2 |  |  | 1111 | 3.347 | 0.5975 | Yes |
| 41 | S100A1 |  |  | 1118 | 3.342 | 0.6094 | Yes |
| 42 | NOLC1 |  |  | 1222 | 3.217 | 0.6135 | Yes |
| 43 | SEMA4D |  |  | 1328 | 3.107 | 0.6171 | Yes |
| 44 | DNTTIP2 |  |  | 1348 | 3.078 | 0.6270 | Yes |
| 45 | TRAF3IP3 |  |  | 1527 | 2.923 | 0.6244 | Yes |
| 46 | BYSL |  |  | 1577 | 2.872 | 0.6313 | Yes |
| 47 | CCL2 |  |  | 1664 | 2.791 | 0.6352 | Yes |
| 48 | COL4A1 |  |  | 1815 | 2.683 | 0.6337 | Yes |
| 49 | MTF2 |  |  | 1858 | 2.643 | 0.6404 | Yes |
| 50 | TCF7 |  |  | 2067 | 2.473 | 0.6338 | Yes |
| 51 | EML4 |  |  | 2092 | 2.457 | 0.6411 | Yes |
| 52 | BPI |  |  | 2121 | 2.442 | 0.6480 | Yes |
| 53 | CNGA1 |  |  | 2164 | 2.410 | 0.6537 | Yes |
| 54 | P2RX5 |  |  | 2332 | 2.305 | 0.6496 | Yes |
| 55 | SSR1 |  |  | 2351 | 2.285 | 0.6567 | Yes |
| 56 | SEC13L1 |  |  | 2697 | 2.093 | 0.6384 | Yes |
| 57 | CYBB |  |  | 2718 | 2.080 | 0.6446 | Yes |
| 58 | PACSIN3 |  |  | 2814 | 2.032 | 0.6449 | Yes |
| 59 | POU2AF1 |  |  | 2861 | 2.006 | 0.6489 | Yes |
| 60 | VRK2 |  |  | 2966 | 1.952 | 0.6482 | Yes |
| 61 | DENND3 |  |  | 2982 | 1.938 | 0.6543 | Yes |
| 62 | HEATR1 |  |  | 3012 | 1.926 | 0.6592 | Yes |
| 63 | C14ORF115 |  |  | 3070 | 1.897 | 0.6619 | Yes |
| 64 | GLRX2 |  |  | 3196 | 1.841 | 0.6593 | Yes |
| 65 | RTCD1 |  |  | 3207 | 1.836 | 0.6653 | Yes |
| 66 | ZNF313 |  |  | 3329 | 1.781 | 0.6628 | No |
| 67 | ATF3 |  |  | 3455 | 1.727 | 0.6597 | No |
| 68 | ZNF267 |  |  | 3725 | 1.614 | 0.6454 | No |
| 69 | CENTG1 |  |  | 4078 | 1.481 | 0.6242 | No |
| 70 | MS4A1 |  |  | 4149 | 1.454 | 0.6243 | No |
| 71 | DDX58 |  |  | 4251 | 1.413 | 0.6219 | No |
| 72 | SLC11A2 |  |  | 4406 | 1.353 | 0.6153 | No |
| 73 | IRF5 |  |  | 4410 | 1.351 | 0.6200 | No |
| 74 | VPREB3 |  |  | 4876 | 1.197 | 0.5893 | No |
| 75 | IGLV6-57 |  |  | 5044 | 1.138 | 0.5809 | No |
| 76 | PIM2 |  |  | 5147 | 1.099 | 0.5773 | No |
| 77 | ECGF1 |  |  | 5392 | 1.016 | 0.5626 | No |
| 78 | ZMYM5 |  |  | 5447 | 0.994 | 0.5622 | No |
| 79 | IGLL1 |  |  | 5555 | 0.957 | 0.5576 | No |
| 80 | RPS24 |  |  | 5699 | 0.911 | 0.5502 | No |
| 81 | ISG20 |  |  | 5742 | 0.898 | 0.5503 | No |
| 82 | ATR |  |  | 5834 | 0.867 | 0.5467 | No |
| 83 | EEF1D |  |  | 6137 | 0.772 | 0.5267 | No |
| 84 | TRIP6 |  |  | 6573 | 0.627 | 0.4961 | No |
| 85 | CXCR3 |  |  | 6719 | 0.581 | 0.4873 | No |
| 86 | IFITM1 |  |  | 7174 | 0.426 | 0.4546 | No |
| 87 | LDOC1 |  |  | 7192 | 0.421 | 0.4548 | No |
| 88 | LOC160313 |  |  | 7321 | 0.375 | 0.4465 | No |
| 89 | PACAP |  |  | 7358 | 0.365 | 0.4452 | No |
| 90 | LRRC59 |  |  | 8052 | 0.087 | 0.3931 | No |
| 91 | FAM50B |  |  | 8137 | 0.061 | 0.3870 | No |
| 92 | ANK3 |  |  | 8438 | -0.052 | 0.3645 | No |
| 93 | OGG1 |  |  | 8672 | -0.158 | 0.3474 | No |
| 94 | PHF20L1 |  |  | 8789 | -0.203 | 0.3394 | No |
| 95 | FAM46C |  |  | 9981 | -0.831 | 0.2525 | No |
Table: GSEA details [plain text format]

  

Fig 2: BRCA\_BRCA1\_POS: Random ES distribution      
 Gene set null distribution of ES for **BRCA\_BRCA1\_POS**
